# Patients’ experience of patient-reported outcomes, continuous feedback, and a solution-focused approach (using DIALOG +) in psychosis care in Sweden

**DOI:** 10.1186/s12888-025-07070-1

**Published:** 2025-07-01

**Authors:** Marcus Lundmark, Katarina Allerby, Andreas Gremyr, Ann-Christine Andersson

**Affiliations:** 1 Department of Psychotic Disorders, Sahlgrenska University Hospital, Gothenburg, Sweden; 2 Jönköping Academy for Improvement of Health and Welfare, School of Health and Welfare, Jönköping, Sweden; 3 Institute of Health and Care Sciences, University of Gothenburg, Gothenburg, Sweden; 4 Department of Care Science, Malmö University, Malmö, Sweden

**Keywords:** Decision making, Shared, Patient participation, Patient reported outcome measures, Problem solving, Psychotic disorders

## Abstract

**Background:**

Involving patients in care and ensuring structured continuous follow-up can be challenging. Using Patient-Reported Outcomes during routine encounters can enable patients and healthcare professionals to collaboratively focus on the patients needs and adjust treatments over time. Previous research highlights the benefits of continuous feedback and solution-focused methods in psychiatric care. DIALOG + is a digitally supported conversational intervention designed to enhance the therapeutic effectiveness through self-reported outcomes and a solution-focused approach. This study explores patients' experiences of using DIALOG + in Swedish psychosis care, focusing on its integration of continuous feedback and a solution-focused approach to support active patient participation in planning and evaluation of care.

**Methods:**

A qualitative exploratory study using Reflexive Thematic Analysis was conducted. Individual semi-structured interviews were carried out with ten patients who had used DIALOG + three or more times.

**Results:**

The analysis identified two themes: *“The supportive features of DIALOG* + *”* and *“DIALOG* + *provides a constructive structure”*. These themes included six sub-categories:* “Expanded the understanding of my health,”; “Moving toward improvement,”; “Provided memory support,”; “Empowering participation,”; “Distinguishing DIALOG* + *as a constructive complement,” and “Experiences of the digital interface.”*

**Conclusion:**

This study suggests that DIALOG + may enhance care for patients with psychotic disorders by:

1. Expanding the understanding of the patient's health by enabling a more comprehensive and nuanced picture of the patient's situation.
2. Promoting improvement by focusing on solution-oriented discussions and concrete actions.
3. Providing valuable memory support for patients, which facilitates follow-up and continuity of care.
4. Strengthening patient involvement in their own care
5. Offering a digital interface that facilitates structured communication between patient and healthcare provider.

This intervention has the potential to enhance patient participation and foster co-production, aligning with national healthcare priorities.

**Supplementary Information:**

The online version contains supplementary material available at 10.1186/s12888-025-07070-1.

## Background

Chronic illnesses, including mental health conditions, represent a significant and growing challenge in healthcare [7]. Severe mental illnesses, particularly psychotic disorders, profoundly impact quality of life [18]. People with the psychotic disorder of Schizophrenia typically experience symptoms such as hallucinations, delusions, and cognitive impairments, sometimes with a need for acute inpatient care. Treatment discontinuation is very common [23], and life expectancy is significantly shorter than for the general population [4]. Effective care for psychosis requires a multifaceted approach integrating medical, psychological, and social support strategies. While patient participation in planning and evaluation of care is legally mandated in Sweden [22], symptom severity and treatment discontinuation can hinder meaningful engagement and a reciprocal dialogue during routine care encounters.

Approaches like person-centred care and patient co-production have emerged as promising strategies to address these challenges [5, 15]. Batalden defines co-production in healthcare as the *"interdependent work of users and professionals who are creating, designing, producing, delivering, assessing, and evaluating the relationships and actions that contribute to the health of individuals and populations"* (2018). This concept highlights the shared roles of patients and professionals in fostering effective health services and achieving better health outcomes. Use of Patient-Reported Outcome Measures (PROMs) and Patient-Reported Experience Measures (PREMs) has potential to enable tailored, person-centred care through systematic feedback [9]. Outcome management is an increasingly preferred method that involves continuous assessment and monitoring of a patient's progress during treatment to positively influence the overall treatment process [11]. This type of continuous, real-time feedback is particularly beneficial in addressing any unexpected challenges and ensuring that the treatment aligns with the patient's evolving needs, which has been shown to be more effective than treatment as usual [8, 12].

DIALOG + is a structured, evidence-based intervention designed to enhance therapeutic effectiveness during routine meetings, and an example of a way of integrating feedback and problem solving at point of care [19]. Patients rate their satisfaction across life domains and treatment aspects, providing a basis for focused, solution-oriented discussions with their clinicians. Previous research highlights the potential of DIALOG + as an effective, low-cost intervention for routine mental health care, particularly for individuals with psychosis. A cluster-randomised trial by Priebe et al. [19] demonstrated significant improvements in quality of life, unmet needs, psychopathological symptoms, and social outcomes, with sustained benefits over 12 months. Further analysis by Priebe et al. [20] confirmed these findings, suggesting a 72% likelihood of cost savings alongside improved outcomes, though longer-term economic evaluations are needed. Complementary studies emphasise DIALOG + 's structured, solution-focused framework that fosters patient empowerment and self-reflection [17]. In a thematic analysis of patient interviews by Priebe et al. [20], DIALOG + seems to empower patients by facilitating structured, meaningful interactions that promote self-reflection, prioritisation of concerns, and collaborative care planning. Additional trials, including an uncontrolled German pilot [6] and a cluster-randomised study in low- and middle-income countries [10], reported improvements in quality of life and specific life domains, though results on clinical symptoms were mixed. Use of DIALOG + has been studied in a growing number of countries, with overall positive results, but no studies have been done in Sweden yet, particularly regarding its capacity to support patient participation in routine psychosis care.

### Aim

This study aims to explore patients' experiences of using DIALOG + in Swedish psychosis care, focusing on its integration of continuous feedback and a solution-focused approach, to support active patient participation in the planning and evaluation of care.

## Method

### Study design

This qualitative exploratory study utilised Reflexive Thematic Analysis [3] to examine patients’ experiences of using DIALOG + in outpatient psychosis care in Sweden. The exploratory design was chosen to provide a nuanced understanding of patients’ perspectives and the practical application of DIALOG + in routine clinical settings.

### The DIALOG + intervention

DIALOG + is an evidence-based, computer-mediated intervention developed to enhance the therapeutic value of routine clinical meetings [19]. It builds on principles of person-centred care, continuous feedback, and solution-focused therapy, as well as insights from quality-of-life research, facilitating collaborative decision-making between patients and clinicians. In this article, the entire DIALOG + session, which includes rating, topic selection, and the solution-focused approach, is referred to as the "intervention”. A DIALOG + application, available on a computer, is used during the session to support the process, further described below.

During a DIALOG + session, patients are invited to rate their satisfaction with eight life domains: mental health, physical health, job situation, accommodation, leisure activities, partner/family, friendship, and personal safety. They also evaluate three treatment aspects: medication, practical help, and meetings with professionals. Additionally, patients identify the areas where they would like further support.

**Fig. 1:**
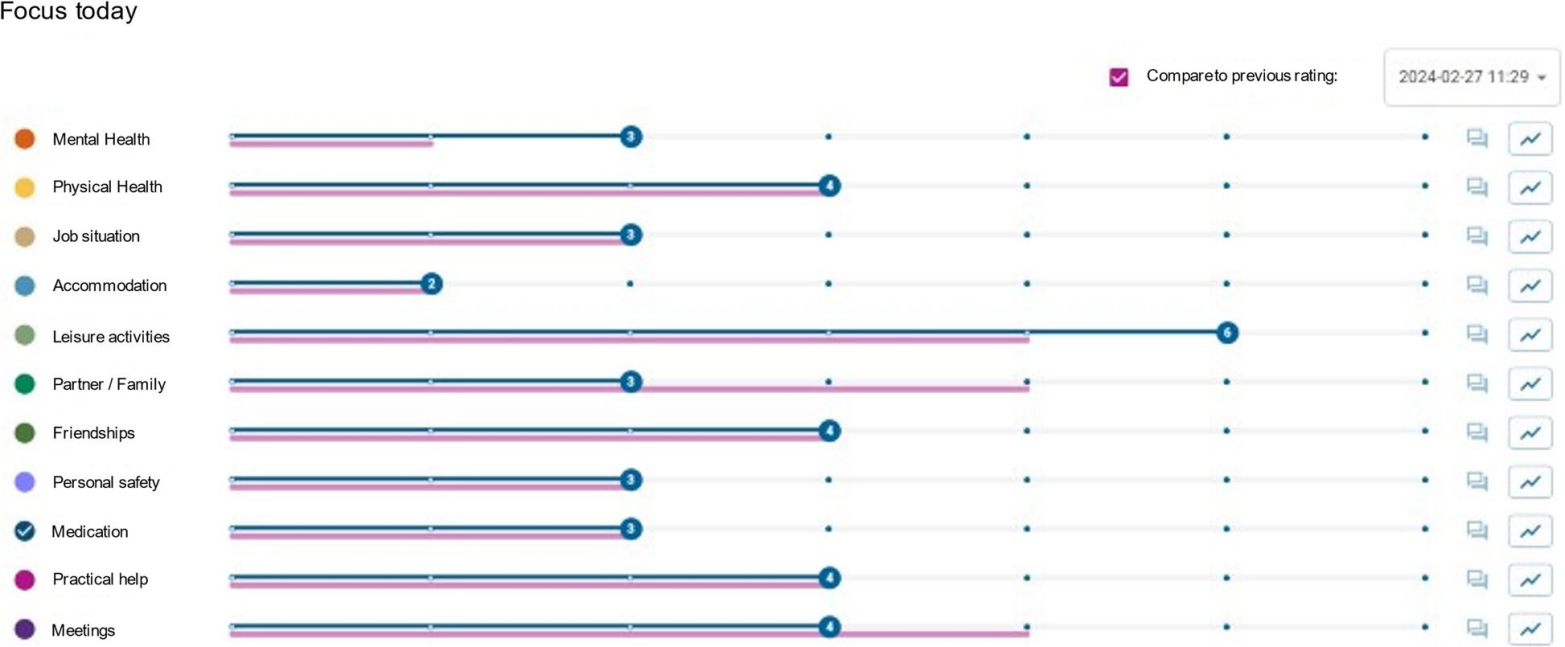
The DIALOG + interface showing patient's ratings, where the current session ratings (top blue line) are compared to a selected previous session's ratings (bottom pink line). At the far left, each category for conversation is shown

Using the overview of ratings, the patient and clinician jointly review concerns and determine which areas to prioritise during the meeting. If DIALOG + sessions have been done previously, current ratings can be compared to earlier ones, offering a longitudinal view of the patient’s progress (Fig. 1). Each selected concern is addressed using a structured, four-step solution-focused approach 1) Understanding: Clarify the patient’s current concerns, 2) Looking forward: Identify scenarios for improvement, 3) Exploring: Discuss potential actions to address concerns, and 4) Agreeing: Decide on concrete actions to improve the patient’s condition and social situation.

At the end of the session, a summary of the ratings and agreed-upon actions is saved and can be printed for the patient. This ensures a tangible record of the session and can remind the patient of agreed-upon actions and progress that has been made so far. The process aims to empower patients to lead discussions that reflect their priorities and preferences, thereby promoting personalised care. Some DIALOG + sessions were conducted via video meetings. In these cases, clinicians used screen-sharing functionality to allow patients to view and engage with their satisfaction ratings, previous session comparisons, and other interactive elements of the DIALOG + application in real-time. The process and structure of these remote sessions closely mirrored those conducted in person. A more detailed description of the DIALOG + session, along with screenshots of the digital interface, can be found in Supplementary File.”

### Study setting

The study took place at the Department of Psychotic Disorders (the Department) at Sahlgrenska University Hospital in Gothenburg, Sweden. The Department comprises seven outpatient clinics dedicated to serving approximately 3,000 patients, who collectively account for approximately 30,000 clinical encounters each year. The outpatient clinics use Case Management in combination with Flexible Assertive Community Treatment (FACT), with interdisciplinary teams of psychiatrists, nurses, specialised psychiatric assistant nurses, social workers, occupational therapists, psychologists, and physiotherapists or health coaches. DIALOG + is seen as an intervention that potentially complements current practices by adding a structured way of making use of continuous feedback and a solution-focused approach during patient-clinician meetings. A project to adapt and develop DIALOG + for Swedish psychiatric settings was initiated in 2020 [13], followed by testing by a group of clinicians from three of the Department's outpatient clinics [14]. In this study, DIALOG + was used both in in-person meetings and in virtual meetings with patients due to the ongoing COVID-19 pandemic at the time.

### Recruitment and participants

Participants were recruited from the three outpatient units that started testing DIALOG + at the Department. Screening was conducted by clinicians familiar with the patients, identifying individuals meeting the inclusion criteria: 1) experience of three or more DIALOG + sessions (to ensure enough experience to provide comprehensive information about DIALOG +) and 2) being in active treatment with at least one clinician meeting every two months. Participants were approached during routine clinical visits by their treating clinicians, who provided information about the study and invited them to participate. Fourteen patients met the inclusion criteria, and all were invited to participate, with three declining and one lacking sufficient Swedish to participate fully in the interview. The final sample consisted of 10 patients, six of whom were women. The overall age range varied from 31 to 63 years. These patients received DIALOG + sessions from seven clinicians (nurses and mental health nurses) with experience working in outpatient care ranging from 2 to 26 years. Nine patients had had contact with their respective outpatient unit for over one year. In the past 12 months, these patients met with their clinicians on average 32 times. Patients were diagnosed with a schizophrenia spectrum disorder (*n* = 8), and mood disorders (*n* = 2). Patients included had used DIALOG + through video meetings only (*n* = 3), in-person meetings only (*n* = 6), and a mix of video and in-person meetings (*n* = 1). All participants provided informed consent prior to their involvement in the study.

### Data collection

Data were collected through individual interviews conducted between December 2021 and January 2023 at the participants’ outpatient clinic (*n* = 7) or via video meetings (*n* = 3). The interviews, lasting 20–45 min (on average 34 min), were conducted by the first author using a semi-structured interview guide adapted from Priebe et al. [20] and translated into Swedish. Participants were asked to describe their experiences of meetings before and after the introduction of DIALOG + , specifically in relation to how topics were addressed, the decision-making processes, and the structured format of the DIALOG + sessions compared to routine encounters. Specific questions explored their experiences with self-rating, the solution-focused approach, and the DIALOG + interface, as well as any changes in the therapeutic relationship and preferences for using DIALOG + in routine care. One question from the original guide concerning the font size in the DIALOG + software was omitted, and a new question was added to invite participants to share additional topics that had not been previously discussed. Given participants' potential challenges with maintaining focus and engaging in extended reasoning, the interviews were designed to be concise and flexible, using a semi-structured format with suggested follow-up questions. This approach allowed tailoring of the interviews to participants' capacities while ensuring the collection of rich and meaningful data. All interviews were audio-recorded with participants' consent, transcribed verbatim, and securely stored in a de-identified format to protect confidentiality.

### Data analysis

The analysis was conducted using Reflexive Thematic Analysis as described by Braun and Clarke [3]. This method was chosen because it aligns with the study's purpose of exploring patterns of meaning in participants' accounts, emphasising depth and thematic variability rather than quantitative measures or the frequency of codes. RTA is designed to interpret both explicit and underlying meanings in textual data, presenting them thematically while emphasising reflexivity and subjectivity throughout the process. The initial coding and theme development primarily focused on a semantic level, capturing participants’ explicit accounts and descriptions of their experiences with DIALOG + . This approach allowed for a systematic organisation and representation of the data as reported by participants. However, the researchers also engaged in reflective discussions to consider latent meanings and underlying patterns in the data. These reflections aimed to explore the broader implications of participants’ accounts, including assumptions and contextual factors influencing their experiences. This dual approach provided a nuanced understanding of the dataset by combining descriptive coding with interpretive depth.

Data management was supported by Nvivo (version 14). The process began with transcript readings, followed by initial open coding. Codes were developed inductively, without preconceived categories, to uncover unexpected themes. This step generated numerous codes, which were then reviewed and refined through discussions between the coders (ML and KA). In the next phase, themes were identified within the dataset. Coding discrepancies and theme development were resolved through discussions until consensus was achieved. Emphasis was placed on thematic consistency and coherence to ensure a representative portrayal of diverse perspectives. Illustrative quotes selected to exemplify each theme were professionally translated into English to preserve the nuances of participants' perspectives. The analysis was conducted collaboratively by ML and KA, from open coding to result presentation. Reflexive practice was central to the analysis, highlighting how assumptions and prior experiences shaped the process. The authors actively engaged in reflexivity, discussing their thoughts, feelings, and preconceptions to identify how these factors influenced interpretation and result development.

Self-reflection ensured transparency and acknowledged the potential impact of the researchers’ perspectives on the analysis. ML is a psychiatric nurse with expertise in psychosis care and holds a master’s degree in quality improvement. KA, also a psychiatric nurse, has a doctorate in psychiatry focused on person-centered psychosis care and works with first-episode psychosis but lacked prior experience with DIALOG + . As an additional layer of reflexivity, AG and AA reviewed the coding and themes, contributing diverse perspectives based on their different backgrounds and experiences with the intervention and study population. AG is a psychologist with a doctorate in learning health systems and has been involved in adapting DIALOG + to Swedish psychiatric settings since its inception. AA is an associate professor in quality improvement with extensive experience in qualitative research. Other measures to ensure trustworthiness were not employed. While member checking could potentially have strengthened the findings by ensuring participants' perspectives were accurately represented, the RTA approach acknowledges that meaning is constructed during the analytic process and inherently shaped by the researchers’ reflexive engagement with the data [2].

## Results

The analysis resulted in two themes: 'The supportive features of DIALOG + ' and 'DIALOG + provides a constructive structure'. The first theme illuminates the participants' experiences in relation to specific features of DIALOG + , while the second theme explores participants’ perceptions of the digital interface and the differences between DIALOG + sessions and traditional routine encounters (Table 1).

**Table 1:** Themes and sub-themes

Theme 1: The supportive features of DIALOG +	Theme 2: DIALOG + provides a constructive structure
Expanded understanding of my health	Distinguishing DIALOG + as a constructive complement
Moving toward improvement	Experiences of the digital interface
Provided memory support	
Empowering participation	

### Theme 1: The supportive features of DIALOG + 

The supportive features of DIALOG + , encompassing the rating and problem-solving approach, seemed to foster a deeper understanding of personal well-being, encouraged actionable planning, and empowered patients to actively participate in their care. Participants’ experiences with these features revealed important sub-categories, such as memory support, empowerment, and facilitating progress toward goals. Through its structured framework, DIALOG + encouraged an exploratory process that contributed to a deeper understanding of personal well-being. This understanding emerged during targeted discussions of key areas, the identification of concrete goals, and the formulation of actionable plans. The iterative design of DIALOG + , incorporating continuous feedback and a problem-solving approach, appeared to foster opportunities for participants to reflect on their progress, revisit challenges, and refine strategies over time. These features seemed to cultivate a sense of control. Memory aids embedded in the process likely enhanced the effectiveness of other features, while the focus on individual choice and participation offered an empowering dynamic that appeared to strengthen engagement. The interaction between the structured elements of DIALOG + and participants’ subjective experiences may have played a role in shaping their perceptions of progress and empowerment.

#### Expanded understanding of my health

Two aspects seemed to help participants achieve a more comprehensive understanding of their health: the rating and the holistic approach provided by the multiple life and treatment domains assessed. Actively rating different health areas gave participants an overview of their overall well-being, highlighting both strengths and areas of concern.

The rating process seemed to serve as a foundation for reflection and discussion, helping participants identify the factors influencing their ratings in different areas. It transformed the abstract notion of "how I feel" into a concrete understanding of the factors impacting their well-being, allowing participants to recognise the various aspects contributing to their overall health.

*It becomes a visualisation ... making my well-being status more concrete. (p8)*

*I felt that with DIALOG*+*, it was more mapped out, the feelings you have and how you behave in different situations. You could see that in the rating; on that particular day, I must have felt a bit worse or a bit better. It's interesting to know what affects such things, that you sometimes feel worse. Being able to look at the different variables was good. (p6)*

Comparing current ratings with previous ones may have contributed to participants’ overall understanding of their well-being and changes over time. The ability to track these changes seemed to provide an additional layer of insight into their perceptions of health, potentially helping them draw conclusions and identify underlying factors influencing their condition.

*It helps to have the overall picture, like how it was then compared to how it is now. (p10)*

*... the function seems good, because then you can clearly see if you have improved in any area. (p2)*

Moreover, the rating feature may serve to acknowledge the potential for change, as participants could explicitly observe improvements or periods of lower satisfaction, which appeared to foster a sense of hope. Some participants also seemed to associate this feature with identifying topics for discussion, suggesting that an expanded understanding of one's health could be an important initial step in determining relevant areas for further exploration and prioritising actions.

*It was interesting that the way you feel could change so much, from feeling very bad at one time to feeling much better the next time. You could identify why you felt like that. Sometimes it could be that something had happened, and sometimes it was just my mood on that particular day. (p6)*

DIALOG + 's inclusion of ratings across 11 different life and treatment domains may have provided participants with a more holistic perspective on their health. This approach seemed to help participants gain insight into areas of their well-being that they might otherwise have overlooked or not considered discussing. Expressions of feeling more respected emerged, as the use of DIALOG + appeared to make the clinician more aware of additional factors affecting her, thereby offering a fuller understanding of her life situation and challenges.

*I think DIALOG*+ *gives a more holistic perspective. You get the whole picture of mental health. And it's everything from social factors, and specifically the psychiatric, if you have any psychotic symptoms or similar. (p10)*

#### Moving toward improvement

Participants described how DIALOG + may have supported them in moving forward by providing the opportunity to select relevant domains for further discussion, identify challenges, and plan actions. Most participants highly valued the ability to choose specific areas for discussion and support. This process appeared to be a straightforward and practical way to pinpoint areas where support was needed. The variety of domains identified seemed to highlight what could be addressed and allowed for the exploration of challenging topics that might not otherwise have been discussed. This enabled participants to engage more efficiently with specific areas.

*It is a very straightforward model. When you have a conversation [before using DIALOG*+*], it takes a while before you start talking about certain things. You may need to meet a few times. This model highlights different areas, that's what I mean by saying it's more straightforward. You choose areas, and then we dig deeper into what you need to talk about. (p3)*

The rating appeared to help participants identify areas requiring attention, and some expressed that this was important for deciding which domains to prioritise during the encounter. Participants reported that reviewing the results of the ratings helped clarify priorities, and some also felt it supported the clinician in maintaining focus and guiding the discussion.

The opportunity to address domains with lower satisfaction levels may have encouraged both participants and clinicians to confront and explore challenging issues that might otherwise have been overlooked.

*It was very concrete that you had to choose which areas you wanted more support and help, that you could bring up a topic and go into it a bit deeper, and gather your thoughts around it. It was helpful to be able to do that. This makes it stricter and more concrete and tackles the problem. Otherwise, it's easy to not talk about what's difficult and avoid it. (p2)*

Several participants described how DIALOG + supported them in pinpointing problems, deciding on actions to address them, or setting and planning goals to achieve desired outcomes. This process appeared to foster a sense of growth, particularly when participants succeeded in reaching their goals. Most participants’ reflections on defining problems or goals and identifying ways to address them seemed closely tied to the solution-focused approach. Participants reported that this approach helped them maintain focus, collaborate on finding solutions, and agree on concrete actions. Some statements suggested that the structured framework of DIALOG + may have facilitated addressing issues that had previously been difficult to resolve. Additionally, the solution-focused approach appeared to encourage some participants to involve others, such as family members, in their support networks and to seek out further care and assistance.

*From the second session, we agreed on various actions, which I call some kind of goals. I've actually reached several of them since then. The next time, we sort of started from scratch, but we also looked back at how things looked before. And then we set new goals. And I think it helps me a lot, like... to find some kind of... well, way forward and some balance in life. (p10)*

*I feel that this [DIALOG*+*] is more concrete because here you find the weaknesses in a completely different way, simpler, I think. That's how it feels to me. It feels like I can get things I'm unhappy with fixed in a more concrete way. (p7)*

Not all participants appeared to experience the same degree of success in agreeing on or implementing actions. Some participants noted that time constraints during the session, specifically the time required to complete the rating process, seemed to limit or even eliminate the time available for discussing the problem and formulating agreed actions. Others indicated that taking action was challenging due to factors such as ill health or other unspecified reasons. However, these instances of difficulty may represent opportunities for initiating a new round of problem-solving and action-planning, potentially leading to more tailored and effective support.

*Sometimes I did what we had agreed on, and sometimes not. The interesting thing is why it turned out that way. (p6)*

#### Provided memory support

Participants described various ways in which DIALOG + supported their memory. The software collects information, such as self-assessments across life and treatment domains, selected discussion topics, and agreed-upon actions from each session. This information is visually presented in subsequent sessions, which seemed to help both patients and clinicians track progress, revisit key points from earlier meetings, and reflect on changes over time. To further support memory, agreed-upon actions can be printed for patients to take home, ensuring they have a tangible reminder of what was discussed. Participants indicated that this memory support was helpful in both concrete tasks, such as selecting topics from a visual list, and more abstract processes, such as developing an understanding of their well-being over time. Regular use of DIALOG + was also noted as beneficial for recalling previous responses and discussions, which may be particularly valuable for patients with short-term memory difficulties.

*I think it can be quite good to get a printout. I get one to remember what we have talked about. And it becomes a way for me to look at and go back to and think about what I should do to achieve my goals. (p10)*

*I think that it's good actually, just that you have... we've been doing it regularly now and so on, it means that you might remember why you answered a certain thing and so on. And then you remember, so it doesn't just disappear, so to speak....from your memory. (p8)*

#### Empowering participation

Participants described how DIALOG + supported their ability to make their own choices during the therapeutic process. Their statements suggested that the opportunity to select topics for further discussion and being encouraged to find their own solutions contributed to feelings of empowerment. Participants appeared to value this autonomy, as it allowed them to take ownership of decisions and generate their own solutions. Some participants highlighted the importance of being the one to make decisions, which seemed to foster a sense of self-confidence and independence. At the same time, participants’ statements indicated that clinicians sometimes needed to take a more active role in problem-solving and guide participants toward identifying the most suitable solutions. Additionally, active involvement in decision-making and topic selection may have enhanced participants’ sense of autonomy and fostered mutual respect within the therapeutic relationship.

*I thought it was very good that the patient independently gets to choose in what areas they want more help or support. I think it's good that people can choose themselves and feel more independent, because when you have a psychotic disorder, you can feel very dependent on others. (p2)*

*I definitely felt that I was involved when I tested DIALOG*+*. I was involved in developing these different parts that were needed to come up with ideas on how to solve my problems. (p6)*

### Theme 2: DIALOG + provides a constructive structure

Participants appeared to distinguish DIALOG + from traditional routine encounters by emphasising its structured yet flexible approach and its potential effectiveness in promoting proactive health management. While participants appreciated the constructive structure provided by DIALOG + , most expressed a preference for the conversational openness found in encounters where DIALOG + was not used. As a result, several participants suggested that combining sessions with and without DIALOG + may optimise their overall healthcare experience. Feedback on the digital interface indicated that it was generally perceived as user-friendly and supportive, although a few participants reported challenges with navigation and design. These findings suggest that DIALOG + may empower patients to take a more active role in managing their health by offering a structured framework for discussions and decision-making while also highlighting the importance of balancing structure with conversational flexibility.

#### Distinguishing DIALOG + as a constructive complement

Through descriptions of the supportive structure of DIALOG + and reflections on how it should be used, this subtheme illustrates how participants view DIALOG + as a constructive complement to routine encounters with their clinician. Participants provided varied accounts of routine encounters that often lacked a standardised structure. These accounts seemed to highlight qualitative differences in such meetings, where the experience was often influenced by the clinician's skills and the therapeutic relationship. Some participants described more traditional, medically focused encounters, where the clinician typically led the conversation and prioritised discussions about medication and symptoms.

*It was different. It was very much about how are your medications going? And how are your symptoms [traditional routine encounters]? And they just focused on that. (p10)*

*When you're mentally ill, the clinician takes over in a way, and I might have had other things that I wanted to talk about. They see the illness, that you don’t see yourself. You talk about things that the clinician wants to talk about. (p4)*

Other participants reflected that during routine encounters (not using DIALOG +), the clinician encouraged them to participate, inviting them to decide on what topics to discuss or taking a more whole-life perspective when asking questions regarding health. When these conversations were described as beneficial, the relationship with the clinician was an important factor, and participants described a good, well-established relationship. Although participants highlighted the importance of a good dialogue, they also highlighted the risk that some topics would be overlooked.

*Previously, I used to get asked whether there was anything special I wanted to address and how different things were working out, for example, my medication. Sometimes you miss things that you don't talk about. (p2)*

*It was more like ‘How are you?’ We talked superficially about whatever came to mind, and then we might forget about different areas. (p4)*

DIALOG + was described as a structured yet flexible approach, contrasting with the free-form nature of traditional routine encounters. Its structured framework seemed to enable a more focused and efficient dialogue while still allowing for in-depth exploration of specific topics. Furthermore, it was seen as a way to ensure that critical areas of concern were addressed in a systematic way. Participants indicated that DIALOG + facilitated dialogue, as it was structured in a way that provided valid questions for discussion and steered the conversation toward the most important topics, especially in the early stages of the therapeutic relationship. Additionally, DIALOG + appears to assist patients in discussing areas they may not otherwise address, thus providing an opportunity for patients to open up more quickly.

*There is a clear difference when we’ve used DIALOG* + *. It's mostly the structure of the conversation. Well, we speak very freely about the topic even when we use DIALOG* + *, but it's still this thing that you have a follow-up, you get to think about the different ratings and things like that. It sort of creates a structure in the conversation and helps maintain focus. (p8).*

DIALOG + was appreciated for its interactive and collaborative nature, offering a platform where patients could develop and share their own ideas while receiving constructive feedback. This aspect was seen as crucial as it helped participants evaluate the feasibility of their ideas and seemed to contribute to a more dynamic and supportive interaction.

*You help each other, and it's very good that there is this room to come up with your own ideas, and at the same time, you can get very good feedback on whether it's constructive or not. (p2)*

Some participants suggested that using DIALOG + earlier could have helped them address key issues sooner. This suggests that using DIALOG + early in the care process could potentially lead to reduced length of therapy.

*I felt I got a lot out of it. It covered important areas and so on, but I would have appreciated if we could have used it a lot earlier. I might have been able to leave certain things behind much sooner. Certain things that might be more difficult to talk about would have been pinpointed in a much earlier stage. (p3).*

Although almost all participants described the usefulness of the structure provided by DIALOG + , several participants stated that traditional routine encounters were also needed. Others suggested that a different structure could be used, where helpful features of DIALOG + (e.g., choosing topics) are used but where the dialogue could then depart from the DIALOG + format, making the conversation more “free” within the DIALOG + session. Others pointed out that the ability to observe changes in health over time or monitor the achievement of established goals requires a timespan of several weeks between DIALOG + sessions, and emphasised the significance of employing DIALOG + over an extended period.

#### Experiences of the digital interface

Participants provided feedback on their experiences with the digital interface. Their statements described their perceptions of and interactions with the user interface, including its design, ease of use, and overall usability. The statements suggest that the participants found the digital interface to be user-friendly and easy to understand. They appreciated the visual presentation of results and felt that it provided valuable support, even for individuals who struggle with the use of technology. Some participants viewed DIALOG + as an effective and accessible self-analysis tool that offered a more comfortable experience than traditional paper-based alternatives. Some downsides were reported. One participant encountered difficulties navigating the digital interface during the rating process, particularly when changing questions or topics and choosing areas for further support, while another participant needed support from the clinician due to limited computer skills. One participant suggested that the design could be more user-friendly and intuitive, with larger icons.

*I think it was a great idea (with the DIALOG*+ *program on a computer) actually, because it's not like staring over a piece of paper and not finding the answer. (p1)*

*I think this seems to be easy to work with. Now I get support from my therapist all the time, because I am so unsure about computers and technology. It seems to be good; I get a good impression of it. Pretty easy to understand for someone who has difficulty understanding computers, so it was easy to understand at the time. (p9)*

Some DIALOG + sessions were conducted via video meetings, during which clinicians used screen-sharing functionality to enable patients to actively view and engage with their ratings, previous session comparisons, and other interactive elements of DIALOG + in real-time. This approach preserved the structured and interactive features of DIALOG + and facilitated patient involvement in a remote setting. Participants generally provided positive feedback on using DIALOG + in video meetings, noting that the screen-sharing functionality enhanced clarity and made these meetings feel more concrete than traditional video meetings not using DIALOG + .

*I think it worked very well using DIALOG*+ *during video meetings. It was perhaps even easier to do it that way than usual. It makes things more concrete. I'm not a very tech-savvy person, so what you see on a screen feels kind of not real, even though I know that the person I'm talking to through the screen feels a bit strange and distant. DIALOG*+ *makes it more concrete when you meet through a screen. (p2)*

## Discussion

### Support for co-production using feedback and a solution-focused approach

This study explored the experiences of patients using DIALOG + in routine psychosis care in Sweden. The results highlight the potential of DIALOG + to foster co-production by supporting patients to actively participate in their care planning and decision-making. The structured format of DIALOG + may support patients to visualise their health status through self-assessments and to select areas for further discussion. This process may support preference-based decisions and facilitate the development of care plans aligned with patients’ goals. The iterative nature of DIALOG + appears to align with Elwyn’s co-production cycle (*co-assess, co-decide, co-design, co-deliver*), helping ensure active patient involvement throughout the care process [5].

Participants seemed to appreciate the continuous feedback provided by the tool, particularly the ability to track progress and reflect on changes over time. This appears to align with the principles of outcome management, which emphasise the importance of real-time feedback in optimising treatment outcomes [8]. By making trends in well-being more visible, DIALOG + may offer opportunities for patients and clinicians to collaboratively identify challenges and explore actionable solutions. Moreover, these ratings may serve as early indicators of improvement or potential deterioration, guiding timely interventions.

Based on our analysis, the structured, solution-focused approach within DIALOG + may enhance its role in co-production by fostering collaborative problem-solving. Patients and clinicians work together to address specific concerns, agree on priorities, and implement targeted actions. This may promote a sense of ownership among patients while helping to maintain focus during clinical encounters, balancing structure with conversational flexibility.

The findings from this study thus appear to align with previous research, suggesting the benefits of continuous feedback and solution-focused interventions in mental health care and its potential to reduce deterioration and improve recovery rates compared to standard care [12]. The present findings appear to resonate with those of Omer et al. [17], who identified that DIALOG + supports structured communication, self-reflection, and a sense of empowerment among patients with schizophrenia or related disorders. Similarly, Matanov et al. [16] found the intervention to be acceptable and helpful in facilitating goal-oriented conversations across clinical settings for patients with chronic depression. The current study may complement and extend the existing literature by providing insights into how DIALOG + may be experienced as supporting collaboration, shared responsibility, and a sense of partnership within routine outpatient psychosis care in Sweden.

### Clinical encounters with and without DIALOG + 

Participants reported that routine encounters without DIALOG + often lacked structure and sometimes overlooked important topics. The use of DIALOG + was described as providing a structure that enabled focused discussions and the exploration of a variety of health domains based on the patient’s needs. In contrast to routine encounters, which were described as highly variable and dependent on the clinician's preferences, competencies, or current state, DIALOG + seems to provide a more standardised framework that may help ensure all patients have the opportunity to discuss what is most important to them. This structure may reduce the influence of individual clinicians' approaches and facilitate a more equitable care process, enabling patients to focus on their priorities regardless of the clinician’s preferences or expertise.

Moreover, the use of DIALOG + was perceived as offering valuable memory support through various features, such as written transcripts, visualisation of results, and consistent follow-up, which seemed to help participants retain, organise, and recall important information. Such cognitive compensatory interventions have been suggested to support improvements in functioning for patients with psychotic disorders [1]. Even though all participants were satisfied with the structure within DIALOG + , some participants highlighted time constraints regarding completing a full DIALOG + session during the visit. Time constraints may partly stem from the functional impairments often experienced by patients with psychotic disorders, such as difficulties with concentration, memory, or processing information. These challenges might make it harder for some patients to engage fully with the structured elements of DIALOG + within the time available. Additionally, the complexity of issues raised during sessions may influence the ability of both patients and clinicians to cover all necessary aspects of care within the limited session duration, potentially requiring additional time during the session or prioritisation of key concerns. Additionally, many participants suggested combining DIALOG + sessions with less structured routine encounters to optimise the healthcare experience. This may underscore the role of DIALOG + as a constructive complement to more free-form or traditional therapeutic encounters.

### Practical implications

Our findings support the theoretical functionality of DIALOG + and previous research, suggesting its potential to facilitate co-production in healthcare by placing the patient in the role of a decision-maker. Furthermore, DIALOG + enables longitudinal tracking of the patient's progress over time, providing a comprehensive view of the patient's well-being and helping support for the evaluation of treatment effectiveness on an individual level. Through the use of continuous feedback and a solution-focused approach, DIALOG + also may help facilitate tailored care plans based on the patient's needs. Beyond using DIALOG + on an individual level, aggregated patient rating data may support the identification of strategies and areas for improvement at various levels within the healthcare system, contributing to quality improvement initiatives that ultimately enhance overall care and outcomes for individuals with severe mental illness.

Participants' experiences of using DIALOG + via video meetings were positive, although only a few participants used the intervention in this format. This is consistent with previous research indicating that remote interventions are generally feasible and well accepted among individuals with schizophrenia-spectrum disorders. However, it is important to note that previous studies on video-based interventions in this patient group have shown methodological limitations and a high risk of bias [21].

### Future research

An ongoing study in psychosis outpatient care settings in Sweden is investigating clinicians' experiences with DIALOG + . The study aims to explore how DIALOG + facilitates patient participation and to identify challenges associated with participation within the context of patient-clinician interactions. Future studies could help explore the relationship between the use of DIALOG + features, such as continuous feedback of patient’s ratings, and the solution-focused approach, and their effects on patients' satisfaction with life areas and the care they receive.

### Limitations

This study was conducted during the early stages of DIALOG + implementation when only a few outpatient units had begun using the intervention. The inclusion criterion required patients to have completed at least three DIALOG + sessions, further limited the participant pool, and the study sample may not represent the broader outpatient population. For instance, patients who did not have a positive initial experience with the intervention or who faced severe cognitive difficulties may have chosen not to continue participating in DIALOG + sessions. Another limitation of this study was the participants' difficulties in maintaining focus and engaging in extended reasoning during the interviews. To address these challenges, the interviews were intentionally designed to be concise, which may have limited the depth of the data collected. Although the interview guide was comprehensive, covering a broad range of questions and topics, the interviews lasted an average duration of 34 min. The semi-structured format, which included suggested follow-up questions and overarching topics, allowed for flexibility in adapting to the participants’ capacities. While this approach ensured that participants could meaningfully contribute, the need to balance conciseness with depth may have constrained the ability to explore certain areas in greater detail.

## Conclusions

This study suggests that DIALOG + may enhance care for patients with psychotic disorders by:

1. Expanding the understanding of the patient's health by enabling a more comprehensive and nuanced picture of the patient's situation.
2. Promoting improvement by focusing on solution-oriented discussions and concrete actions.
3. Providing valuable memory support for patients, which facilitates follow-up and continuity of care.
4. Strengthening patient involvement in their own care
5. Offering a digital interface that facilitates structured communication between patient and healthcare provider.

This intervention has the potential to enhance patient participation and foster co-production, aligning with national healthcare priorities. These conclusions are based on a qualitative study involving ten participants. While the findings are promising, they should be interpreted cautiously. Future studies could examine how DIALOG + features, like continuous patient feedback and a solution-focused approach, influence patients' satisfaction with life and care.

## Supplementary Information

Supplementary Material 1.

Supplementary Material 2.

Supplementary Material 3.

## Data Availability

To maintain participant confidentiality, per information provided in the informed consent, study data is not publicly available. Reasonable requests to the corresponding author will be considered and a confidentiality assessment will be performed at each individual request.
