# Supplementary material for: Patients’ experience of patient-reported outcomes, continuous feedback, and a solution-focused approach (using DIALOG +) in psychosis care in Sweden
Source: BMC Psychiatry. 2025 Jul 1;25:620. doi: 10.1186/s12888-025-07070-1 (PMC12210646; doi:10.1186/s12888-025-07070-1)
Supplement: Supplementary file 1 — Supplementary Material 1. [file 12888_2025_7070_MOESM1_ESM.docx]

**Description of the DIALOG+ intervention:**

During a DIALOG+ session, patients are invited by clinicians to rate their satisfaction with eight life domains (e.g., mental health, physical health, job situation) and three treatment aspects (e.g., meetings with professionals and medication), highlighting areas where they need further support:


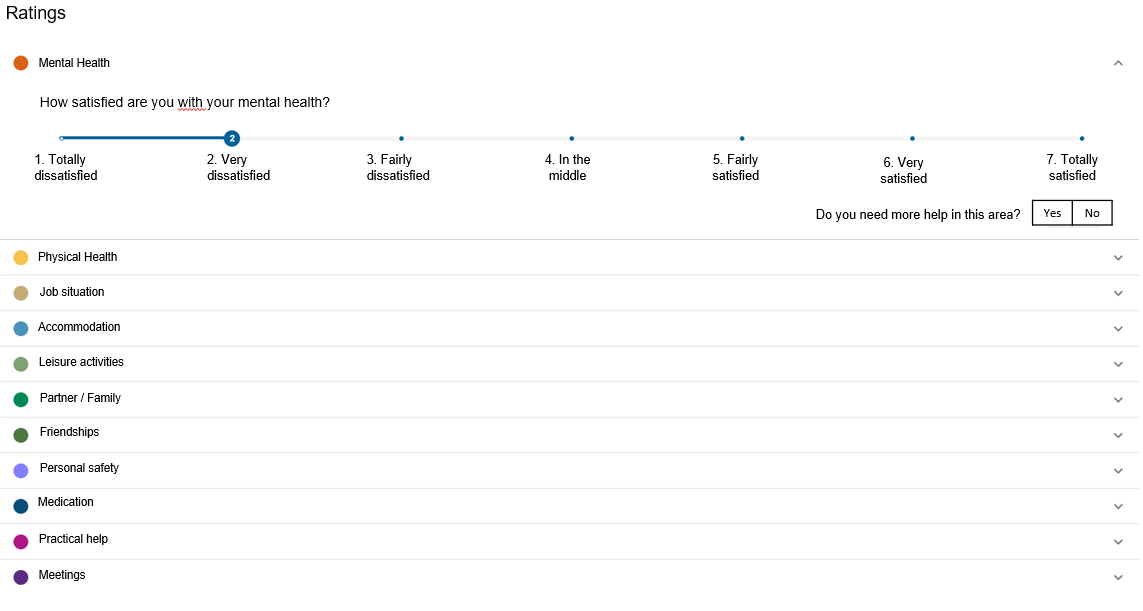
If DIALOG+ has been used before, current ratings can also be compared with previous ratings, providing a longitudinal perspective on the patient's progress. During the rating process, the patient also selects areas where further support is needed. When looking at the overview of today's rating, the patient and the clinician try to briefly understand the patient's current concerns and which of these should be focused on during the meeting:


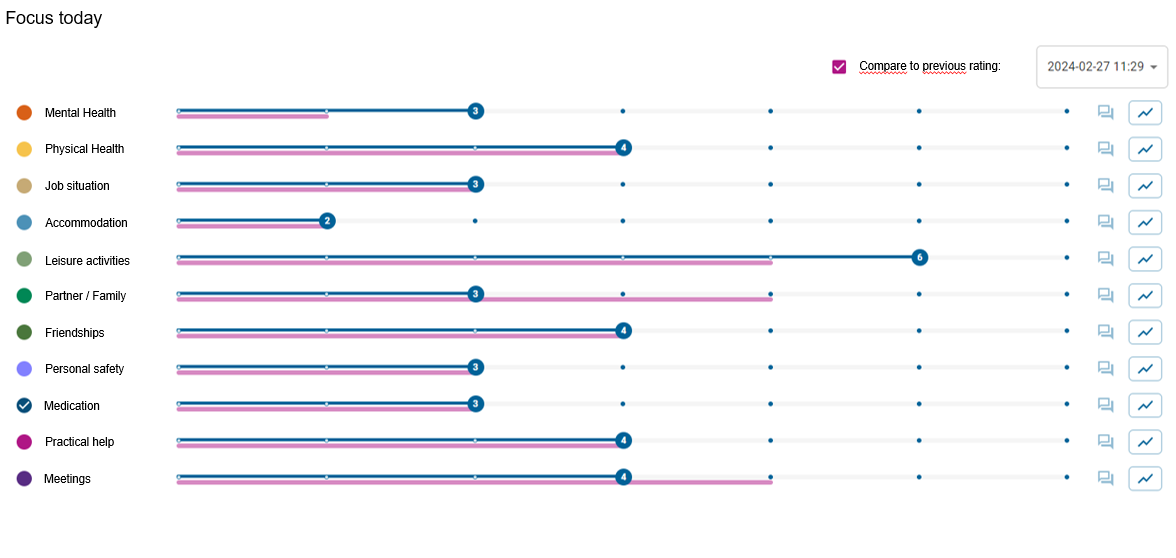
Concerns in each area are addressed in a 4-step solution-focused approach based on solution-focused therapy. The approach is intended to help patients and clinicians to understand the patient's concerns (‘understanding’), identify scenarios for improvement (‘looking forward’), explore options for actions (‘exploring’), and finally agree on actions for improving the patient's condition and social situation (‘agreeing’):


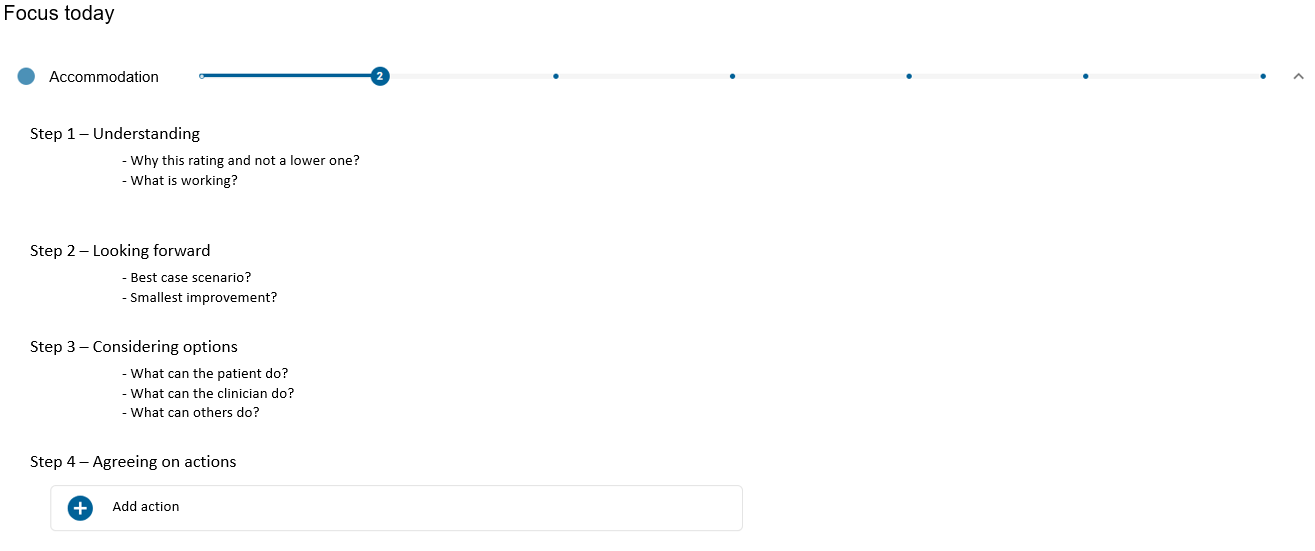


Finally, a summary of the DIALOG+ session is saved in the program. This summary, which includes the patient's self-assessed well-being and agreed-upon actions, can be printed for the patient to take home. At the next DIALOG+ session, agreed-upon actions, along with new ratings, ensure ongoing patient feedback on the care process.
