# Supplementary material for: Patients’ experience of patient-reported outcomes, continuous feedback, and a solution-focused approach (using DIALOG +) in psychosis care in Sweden
Source: BMC Psychiatry. 2025 Jul 1;25:620. doi: 10.1186/s12888-025-07070-1 (PMC12210646; doi:10.1186/s12888-025-07070-1)
Supplement: Supplementary file 2 — Supplementary Material 2. [file 12888_2025_7070_MOESM2_ESM.docx]

Interview guide on patients’ experiences of DIALOG+

1. **(WARM-UP):**

- How long have you been in contact with psychiatric care/your therapist/how many Dialog+ sessions have you taken part in?

1. **(FOCUS): WHAT MEETINGS WERE LIKE BEFORE DIALOG+:**

- As part of this research, you have used Dialog+, where you and your therapist, among other things, assessed how you're doing using a computerised program. Before discussing that, can you describe how your meetings used to be before you took part in Dialog+ sessions?

*(Follow up questions)*

- Can you talk me through how the meeting would usually pan out?
- How did you and your therapist usually decide on what to discuss?
- What would you usually talk about?
- Did you get a chance to talk about the topics that were most important to you?

1. **(FOCUS): WHAT MEETINGS WERE LIKE USING DIALOG+ - GENERAL:**

- Some time ago, you tried Dialog+, where you, among other things, had to rate your well-being and discuss different areas with the help of a computer. Can you tell me how that was?

*(Follow up questions)*

- Can you describe how you and your therapist used Dialog+?
- In what way was it different compared to your previous meetings with your therapist?
- Did you get a chance to discuss what was most important to you?
- Did you find this way of working helpful or not?
- If you could choose, would you prefer Dialog+ or the conversations the way you had them before?

1. **(FOCUS): SELF-RATING OF WELL-BEING AND EXPERIENCE OF CARE:**

- How was it to rate yourself on a scale from 1 to 7, for example, in areas like "mental health," "physical health," "job situation," etc.?

*(Follow up questions)*

- Was it easy or difficult?
- Were there any advantages/disadvantages to doing it this way?
- When you tested it, did you feel you got used to regularly filling in your ratings?
- Did you find it repetitive or valuable to be able to follow a structure?
- For each area you rated, did you get to indicate if you needed more help in that area? Was it helpful for you to fill in what you thought you needed more help with?

1. **(FOCUS): DECIDING ON TOPICS FOR FURTHER DISCUSSION:**

- After you rated the areas, what happened next?

*(Follow up questions)*

- Can you talk me through how it would usually go?
- Did you decide on areas to discuss in more detail later?
- How did you choose which topics to talk about?
- Did you compare your rating to previous ones? If yes, did you find it helpful or not?

1. **(FOCUS): FOUR-STEP APPROACH TO DISCUSSING PROBLEMS – GENERAL:**

- After selecting some areas you wanted to discuss, what did you do then?

*(Follow up questions)*

- Can you describe how you usually proceed?
- Can you remember an area you chose and describe what you and your therapist discussed?
- Can you describe what kind of questions your therapist asked?
- Did you follow any structure or different steps together?
- Did you find it different to talk about things this way compared to your conversations before trying Dialog+?
- Were the discussions helpful for you?

1. **(FOCUS): FOUR-STEP APPROACH FOR DISCUSSING PROBLEMS – SPECIFIC:**

- Step 1: Can you tell me in a bit more detail about how you discussed problems?
- Step 2: Can you describe how you discussed improving your situation?
- Step 3: How did you discuss what could be done to improve your situation? And who would help?
- Step 4: How did you finish up talking about the topic?
- Did you come to any decisions or some sort of action plan?
- How did you agree on which decisions or actions to take?
- Did you review this plan at the beginning of the next session?

1. **(FOCUS): DIALOG+ SOFTWARE:**

- Did you use a computer that you looked at together, or was it mostly your therapist who used the computer?
- What did you think of the computer program?
- How did you feel about the colours used in the program?
- Is there something you missed in the program that could be clarified or changed?

1. **(FOCUS): OVERALL THOUGHTS/EXPERIENCES OF USING DIALOG+:**

- If you were to summarize your impression of using Dialog+ in conversations with your therapist, what would you say?
- Has this way of conversing made any difference for you? Has it affected your life in any way, big or small?
- Has your relationship with your therapist changed in any way?
- Would you like to continue using Dialog+ regularly or not?
- Is there anything we have not discussed that you would like to add?
